# Supplementary material for: Cassava Breeding and Cultivation Challenges in Thailand: Past, Present, and Future Perspectives
Source: Plants (Basel). 2024 Jul 10;13(14):1899. doi: 10.3390/plants13141899 (PMC11280297; doi:10.3390/plants13141899)
Supplement: Supplementary file 1 [file plants-13-01899-s001.zip › plants-3055657-supplementary.pdf]

Table S1. Wild *Manihot* species modified from [1, 2]

Manihot species	Geographical origin	Traits	Status
M. tristis Mueller von Argau [1]	Brazil, Venezuela, Suriname	source of protein	Endangered
M. pilosa Pohl [1]	Brazil	Highly related to *M. esculenta*	Endangered
M. leptopoda (Mueller von Argau) Rogers & Appan [1]	Brazil	Adaptive in sandy region	Endangered
M. alutacea Rogers & Apan [1]	Brazil	Adaptive to soil rich in Calcium	Endangered
M. fruticulosa (Pax) Rogers & Appan [1]	Brazil	Edible roots	Medium risk to extinction
M. pentaphylla Pohl [1]	Brazil, Paraguay	Tolerant to drought and soil rich in Calcium	Medium risk to extinction
M. gracilis Pohl [1]	Brazil, Paraguay	Low HCN content	Abundant
M. anomala Pohl [1]	Brazil, Paraguay, Peru, Bolivia, Argentina	enlarged roots, tolerant to drought and soil rich in Calcium	Abundant
M. glaziovii Mueller von Argau [1]	Brazil	High production of latex, resistant to cassava mosaic and brown streak disease	Abundant
M. pseudoglaziovii Pax & K. Hoffmann [1]	Brazil	Minor latex production	Abundant
M. dichotoma Ule [1]	Brazil	Tolerant to drought, rapid stem growth, high carotenoid, protein and minerals in leaf	Abundant
M. procumbens Mueller von Argau [1]	Brazil, Paraguay	Tolerant to Magnesium toxicity	Abundant
M. reptans Pax [1]	Brazil	Tolerant to soil rich Calcium	Abundant
M. stipularis Pax [1]	Brazil	Tolerant to soil rich Calcium	Medium risk to extinction
M. oligantha Pax [1]	Brazil	High protein, enlarged roots	Endangered
M. peltate Pohl [1]	Brazil	Tolerant to soil toxicity	Endangered
M. neusana Nassar [1]	Brazil	Source of apomixis gene	Almost extinted
M. caerulescens Pohl [1]	Brazil, Paraguay	Very tolerant to drought	Abundant
M. leptophylla Pax [1]	Brazil, Ecuador, Peru	Tolerant to *Xanthomonas manihotis*, able to hybridize with cassava	Abundant
M. pringlei Watson [1]	Mexico	Low HCN content	Endangered

Manihot species	Geographical origin	Traits	Status
M.aesculifolia (Humbolt, Bonpland & Kunth) Pohl [1]	Mexico, Honduras, El Salvador, Nicaragua, Costa Rica, Panama	Edible enlarged roots, able to hybridize with cassava	Abundant
M. angustiloba (Torrey) Mueller von Argau [1]	Mexico, USA	Strong odor of HCN	Abundant
M. subspicata Rogers & Appan [1]	Mexico	Strong odor of HCN	Abundant
M. foetida (Humbolt, Bonpland & Kunth) Pohl [1]	Mexico	Edible seeds	Endangered
M. carthaginensis (Jacquin) Mueller von Argau [1]	West Indies, Colombia, Venezuela, Trinidad, Tobago	Tolerant to drought	Abundant
M. esculenta ssp. *Flabellifolia* [2]	Brazil (probably domesticated in the seasonal forests of the Guyanas and Venezuela or in Central Brazil)		
M. esculenta ssp. *Peruviana* [2]	Brazil, Peru		
M. grahami [2]	Brazil, Paraguay, Uruguay, Argentina	Weedy, rapid colonized, adaptive to marginal stressful environments	
M. flabellifolia [2]	Brazil, Paraguay, Uruguay, Argentina		
M. pruinosa [2]	Brazil		
M. saxicola [2]	Guiana, Surinam, Venezuela		

References

1. Nassar, N.M.; Hashimoto, D.; Fernandes, S. Wild Manihot species: Botanical aspects, geographic distribution and economic value. *Genet. Mol. Res.* **2008**, *7*, 16–28. https://doi.org/10.4238/vol7-1gmr389.
2. Lebot, V. *Tropical Root and Tuber Crops: Cassava, Sweet Potato, Yams and Aroids*; Crop Production Science in Horticulture. No. 17; CABI Publishing: Wallingford, UK, 2009; 413p.

Table S2. Cassava varieties registered or released in Thailand

Variety name	Abbreviation	Parents	Year registered or released	Owner/ Breeder	Root yield*(t/ha)	% starch content*	References
Hanatee	HNT	Landrace	-	-	12.1	16.5	[1]
Rayong 1	R1	Landrace	1975	RYFCRC	20.1	18.3	[2]
Rayong 3	R3	MMex55 × MVen307	1983	RYFCRC	17.1	23-28	[2]
Rayong 2	R2	MCol113 × MCol22	1984	RYFCRC	25.6	18.3	[2]
Rayong 60	R60	MCol1684 × Rayong 1	1987	RYFCRC	26.3	20-25	[2]
Sriracha 1	SRC1	MKU2-162 × Rayong 1	1990	Agron-KU	20.1	21.9	[2]
Rayong 90	R90	CMC76 × V43	1991	RYFCRC	23.8	24-29	[2]
Kasetsart 50	KU50	Rayong 90 × Rayong 1	1992	Agron-KU	32.5	24.9	[2]
Rayong 5	R5	27-77-10 × Rayong 3	1994	RYFCRC	27.5	23-27	[2]
Rayong 72	R72	Rayong 1 × Rayong 5	1999	RYFCRC	31.9	20-24	[2]
Huay Bong 60	HB60	Kasetsart 50 × Rayong 5	2003	TTDI, KU	36.3	25.1	[2]
Rayong 7	R7	CMR30-71-25 × OMR29-20-118	2005	RYFCRC	38.1	23-29	[2]
Rayong 9	R9	CMR31-19-23 × OMR29-20-118	2006	RYFCRC	30.6	24-31	[2]
Huay Bong 80	HB80	Kasetsart 50 × Rayong 5	2008	TTDI, KU	34.4	27.3	[2]
Rayong 11	R11	Rayong 5 × OMR29-20-118	2010	RYFCRC	29.8	26-32	[2]
Rayong 84-13	R13	Kasetsart 50 × Rayong 11	2013	RYFCRC	28.2	26.3	[2]
Pirun 1	PR1	Huay Bong 60 × Hanatee	2014	NSTDA, MU	41.3	28.7	[2]

Variety name	Abbreviation	Parents	Year registered or released	Owner/ Breeder	Root yield*(t/ha)	% starch content*	References
Pirun 2	PR2	Huay Bong 60 × Hanatee	2015	NSTDA, MU	36.3	24.7	[2]
Kasetsart 72	KU72	Rayong 5 × OMR29-20-118	2015	Lop KU	30.3	27	[2]
Huay Bong 90	HB90	MKUC34-114-235 open-pollinated	2017	TTDI, KU	31.8	25.7	[2]
Pirun 4	PR4	Huay Bong 60 × Hanatee	2019	NSTDA, MU	35.9	25.4	[2]
Huay Bong 100	HB100	MKUC34-114-235 open-pollinated	2022	TTDI, KU	40.9	25.4	[3, 4]
Chakangrao 2	-	Hanatee x wild sp.	2022	NTTA	31.3	28-30	[4]
HBWX 09-1041-6 (waxy germplasm)	-	SM3375-111 x GM1562-31	2024	TTDI, KU	N.A.	12.3	[4]
HBWX 09-989-9 (waxy germplasm)	-	SM3375-95 x GM1853-85	2024	TTDI, KU	N.A.	14.1	[4]
HBWX 09-826-2 (waxy germplasm)	-	GM1853-25 x GM3375-97	2024	TTDI, KU	N.A.	17.8	[4]
HBWX 09-754-16 (waxy germplasm)	-	GM1791-52 x GM1562-19	2024	TTDI, KU	N.A.	19.9	[4]
HBWX 09-635-4 (waxy germplasm)	-	GM1791-27 x GM1564-85	2024	TTDI, KU	N.A.	15.8	[4]
HBWX 09-612-18 (waxy germplasm)	-	GM1791-17 x GM1853-85	2024	TTDI, KU	N.A.	11.0	[4]
HBWX 09-562-19 (waxy germplasm)	-	GM1791-8 x GM1597-27	2024	TTDI, KU	N.A.	9.5	[4]

Variety name	Abbreviation	Parents	Year registered or released	Owner/ Breeder	Root yield*(t/ha)	% starch content*	References
HBWX 09-317-6 (waxy germplasm)	-	GM1564-59 x GM1853-2	2024	TTDI, KU	N.A.	14.8	[4]
HBWX 09-19-2 (waxy germplasm)	-	GM1562-5 x GM1597-5	2024	TTDI, KU	N.A.	17.2	[4]

*root yield and starch content were retrieved from different sources of references.

List of abbreviation: RYFCRC = the Rayong Field Crops Research Center; Agron-KU= Department of Agronomy, Faculty of Agriculture, Kasetsart University; NSTDA= National Science and Technology Development Agency; MU= Mahidol University; TTDI= Thai Tapioca Development Institution; Lop KU= Lopburi Research Station, Faculty of Agriculture, Kasetsart University; NTTA= Northern Thai Tapioca Association

References

3. Chaengsee, P.; Kongsil, P.; Siriwong, N.; Kittipadakul, P.; Piyachomkwan, K.; Petchpoung, K. Potential yield and cyanogenic glucoside content of cassava root and pasting properties of starch and flour from cassava Hanatee var. and breeding lines grown under rain-fed condition. *Agric. Nat. Resour.* **2020**, *54*, 237–244.
4. Malik, A.I.; Kongsil, P.; Nguyễn, V.A.; Ou, W.; Sholihin, S.P.; Sheela, M.; Becerra López-Lavalle, L.A.; Utsumi, Y.; Lu, C.; Kittipadakul, P.; et al. Cassava breeding and agronomy in Asia: 50 years of history and future directions. *Breed. Sci.* **2020**, *70*, 145–166. https://doi.org/10.1270/jsbbs.18180.
5. Rojanaridpiched, C. (Kasetsart University, Bangkok, Thailand); Thai Tapioca Development Institute (Bangkok, Thailand). Personal communication, 2022.
6. Depertment of Agriculture. Plant Vareities Protection Office. Available online: https://www.doa.go.th/pvp/?page_id=93 (accessed on 3 May 2024).
